# Exploration of Ginkgo biloba leaves on non-small cell lung cancer based on network pharmacology and molecular docking

**DOI:** 10.1097/MD.0000000000037218

**Published:** 2024-03-01

**Authors:** Mingxiao Wang, Ruochen Li, Moiuqi Bai, Xun Zhou

**Affiliations:** a Respiratory Department, The Second Affiliated Hospital of Guizhou University of Traditional Chinese Medicine, Guiyang, China.

**Keywords:** Ginkgo biloba leaves, molecular docking, network pharmacology, non-small cell lung cancer

## Abstract

**Background:**

Pharmacological studies have found Ginkgo biloba leaves have the effect of inhibiting neoplasms, it is clinically used in treating various neoplasms. However, the mechanism of Ginkgo biloba leaves in treating non-small cell lung cancer (NSCLC) remains unclear.

**Methods:**

The active components and corresponding targets of Ginkgo biloba leaves were obtained from the traditional Chinese medicine systems pharmacology database and analysis platform (TCMSP) database, and the targets of NSCLC were obtained from the GeneCards, OMIM, TTD, and DrugBank databases. The common targets of NSCLC and Ginkgo biloba leaves were obtained from VENNY 2.1.0. The STRING database was utilized to construct protein-protein intersections, by using the Cytoscape 3.7.1 software, the protein-protein intersection was optimized and the drug-disease network diagram was constructed. The DAVID database was utilized to perform GO and KEGG analysis. Finally, The Autodock Vina software was used to perform molecular docking of core components and targets.

**Results:**

The key components of Ginkgo biloba leaves in treating NSCLC include quercetin, luteolin, and kaempferol, which may act on Tp53, AKT1, and TNF. Bioinformatic annotation analysis results suggest that Ginkgo biloba leaves may implicated in PI3K-AKT and MAPK signaling pathways. The molecular docking results show the firm affinity between key ingredients and targets.

**Conclusion:**

The potential mechanism of Ginkgo biloba leaves in treating NSCLC has been discussed in this study, which provides a theoretical basis for the clinical treatment of NSCLC and further experimental validation.

## 1. Introduction

Lung cancer is a malignant tumor originating from the mucous membranes or glands of the bronchi of the lungs, it is one of the most common cancers and the leading cause of cancer-related deaths. One statistic shows that in 2020, there were 480,000 new cases of lung cancer diagnosed in Europe, of which lung cancer caused 380,000 deaths.^[1]^ According to the histopathological characteristics, lung cancer can be categorized into two major groups, including small-cell lung cancer (SCLC) and non-small cell lung cancer (NSCLC), SCLC is a poorly differentiated and high-grade neuroendocrine neoplasm and accounts for 10% to 15% of lung cancers, NSCLC can be categorized into adenocarcinomas, large cell carcinomas, and squamous cell carcinoma, large-cell carcinoma, and squamous cell carcinoma, and accounts for the majority of all lung cancers.^[2]^ Most patients with early-stage NSCLC miss the best opportunity for treatment due to a lack of clinical presentations.^[3]^ Currently, the main treatment modalities for NSCLC include surgery, chemotherapy, radiotherapy, immunotherapy, traditional Chinese medicine (TCM), etc. Though targeted therapy and immunotherapy for NSCLC have made great progress in the past two decades, the median overall survival for those metastatic NSCLC unfortunately still remains less than 3 years.^[4]^

In recent years, Chinese medicine treatment for NSCLC has greatly attracted many researchers’ attention and many herbs have been found to exert significant inhibiting effects on lung cancers. Traditional Chinese medicine therapy of cancer has unique advantages such as low toxicity, cheapness, etc. Pang et al found that Dendrobium officinalis has a significant inhibiting effect on the nude mouse tumor model.^[5]^ Wu et al found that Astragalus polysaccharide has a significant antitumor effect on A549 and NCI-H358 cells, which were associated with the down-regulation of p65 and p50 expression, the inhibition effect on transcription activity of NF-κB, and reduction of CyclinD1 and Bcl-xL protein expression.^[6]^ Duan et al found that ginsenoside Rk3 can inhibit cell proliferation and colony formation, block the cell cycle, suppress angiogenesis in H460 cells, and have a characteristic of low toxicity, which can be an effective agent against NSCLC.^[7]^

Ginkgo is a deciduous tree of the Ginkgo family that is highly utilized for its leaves, seeds, and exocarp, it contains a large number of active ingredients.^[8]^ Modern pharmacological studies have found that ginkgo biloba has anticancer, antioxidant, anti-inflammatory, and immunomodulatory effects, and is clinically used in the treatment of respiratory diseases.^[9]^ Han et al found that Ginkgo biloba exocarp extracts can inhibit tumor angiogenesis in lung cancer;^[10]^ Ahmed et al showed that ginkgo biloba can be used in the treatment of hepatocellular carcinoma in rats;^[11]^ Qian et al found that Ginkgo biloba extract exerted antitumor effects on gastric cancer by inhibiting the progression of the cell cycle in gastric cancer.^[12]^ The above studies suggested that Ginkgo biloba may be a potential antitumor agent in treating NSCLC.

Network pharmacology is a new discipline aimed at analyzing the biological network systematically based on systems biology, almost all Traditional Chinese Medicine and World Ethnomedicine play a therapeutic role in disease through multiple molecules, it spans traditional and modern medicine, and can reduce the cost of drug development, it is a powerful tool for discovering active substances and revealing the pharmacological mechanisms of Chinese medicines systematically.^[13]^ Combined with network pharmacology techniques, the potential mechanism of some prescriptions of TCM like Fuzheng Yugan Mixture,^[14]^ commonly used Chinese medicine pairs like Astragalus membranaceus and Angelica sinensis,^[15]^ and single components such as luteolin^[16]^ for the treatment of some common diseases have been revealed, providing new ideas for the treatment of diseases. Recently, researchers have conducted a network pharmacology analysis of TCM to uncover the mechanism of TCM on NSCLC, by using network pharmacology techniques, Ye et al^[17]^ revealed the mechanism of Sophora davidii (Franch.) Skeels flower extract against NSCLC via promoting cell apoptosis by regulating the PI3K-AKT signaling pathway. Jin et al^[18]^ disclosed the molecular mechanism of triptonodiol in the treatment of NSCLC by network pharmacology. Compared with the past research on single-target and single-pathway in TCM, network pharmacology can reveal the mechanism of action of TCM more comprehensively.

Though some well-known clinical experts in China like Professor Zhu frequently use Ginkgo biloba leaves in treating lung cancer, however, the molecular mechanism of Ginkgo biloba leaves in the treatment of NSCLC is still unclear. Therefore, the present study applied network pharmacology to explore the potential mechanism of Ginkgo biloba leaves on NSCLC, which provides strong evidence for Ginkgo biloba leaves in the development of new anti-NSCLC drugs.

## 2. Materials and methods

### 2.1. Screening of ingredients and potential targets in Ginkgo biloba leaves

The traditional Chinese medicine systems pharmacology database and analysis platform (TCMSP) database is an efficient systems pharmacology platform that aims to accelerate drug discovery from herbal medicines, it contains more than four hundred Chinese medicines commonly used in clinical practice, some characteristics of the drug include ADME properties (Absorption, Distribution, Metabolism, Excretion) like drug-likeness, human oral bioavailability, and corresponding targets of drugs is shown in that database.^[19]^ Oral bioavailability (OB) is the extent of a drug’s entry into the body’s blood circulation through oral intake and is a key factor affecting a drug’s ability to exert its normal therapeutic effects, drug-likeness (DL) properties refer to the similarity of a compound to known drugs, thus, the ingredients and corresponding targets of Ginkgo biloba leaves were collected from the TCMSP database with ADME screening with the criteria OB ≥ 30% and DL ≥ 0.18.

### 2.2. Screening the potential targets of NSCLC

Target discovery is essential in drug development, the therapeutic target database (TTD) was expected to facilitate the exploration of the targets of new drugs;^[20]^ OMIM is a database of genes and gene phenotypes and the relationships between them, its main purpose is to collect and curate human genes, traits, and genetic disorders;^[21]^ GeneCards is a comprehensive database that provides extensive information about human genes, it mined and integrated more than 80 data sources;^[22]^ DrugBank database includes more than 6000 investigational drugs, it supplies us with detailed drug and drug–target for approved and experimental drugs;^[23]^ Therefore, we choose the above databases to gain crucial targets of NSCLC, and then the duplicate targets are removed.

### 2.3. The common targets between Ginkgo biloba leaves and NSCLC

The common targets of Ginkgo biloba leaves and NSCLC were obtained from Venny 2.1.0 (http://bioinfogp.cnb.csic.es/tools/venny/index.html). Then, the intersection targets between Ginkgo biloba leaves and NSCLC were acquired.

### 2.4. Construction of drug–compound–target network

The targets of Ginkgo biloba leaves against NSCLC were obtained by using the Venny 2.1.0 online tool, the targets unrelated to NSCLC were eliminated, and then, two files were constructed and imported into the Cytoscape3.7.1 software, and topology analysis was performed. In this figure, different shapes were utilized to describe the drug, the components of the drug, and gene targets.

### 2.5. Construction of protein-protein interaction (PPI) network

Proteins are widely distributed in living organisms and participate in a wide range of life activities like exchanging reaction products and participating in signal relay mechanisms, and the relationships between proteins are exceedingly complex, protein networks are essential for modern life science, the STRING database collects and integrates known and predicted protein–protein association data in organisms.^[24]^

### 2.6. GO and KEGG analysis

The DAVID database is a bioinformatics resource system utilized to perform functional annotation and enrichment analyses of gene lists.^[25]^ Then, gene ontology (GO) enrichment analysis and Kyoto Encyclopedia of Genes and Genomes (KEGG) pathway analysis were performed to understand the connections between genes by that database.

### 2.7. Molecular docking

Firstly, the 2D structure of the ligands in SDF format was obtained from the PubChem database (https://pubchem.ncbi.nlm.nih.gov/), which was transformed into the 3D structure using the Chem3D software, and the structures were optimized according to the minimum energy to obtain the most stable molecular structure. Then, the receptor structures in PDB format were obtained from the Protein Sequence Database (https://www.rcsb.org), then, excess chains, ions, and water molecules were eliminated by the PyMOL software. Afterward, the AutoDockTools software was used to add hydrogen atoms in receptors and convert the format of ligands and receptors. Finally, the molecular docking was performed by the AutoDock Vina software, the PyMOL software was used to visualize the molecular docking results.

## 3. Results

### 3.1. Potential components of Ginkgo biloba leaves

The active compounds of Ginkgo biloba leaves were retrieved from the TCMSP database, and OB ≥ 30% and DL ≥ 0.18 were used as inclusion criteria, then, a total of 27 components and 520 targets were acquired, after eliminating duplicate values of gene targets, and a total of 229 non-duplicate gene targets were acquired.

### 3.2. Main targets of NSCLC

A total of 853 crucial gene targets were acquired in the GeneCards database with a relevance score ≥ 50, 97 NSCLC-related targets were obtained from the TTD database, 469 NSCLC-related targets were obtained from the OMIM database, 52 targets related to some clinically used anti-NSCLC drugs like Dacomitinib and Etoposide. After merging the gene targets acquired from the above databases and eliminating duplicate values, we finally obtained 1348 non-duplicate disease targets.

### 3.3. Targets and ingredients of Ginkgo biloba leaves in treating NSCLC

**Figure 1.**
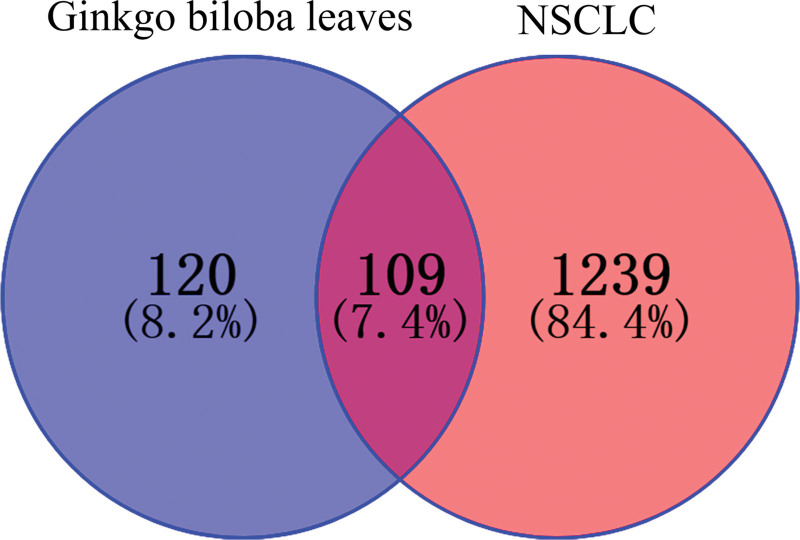
Screening of potential targets of NSCLC with Ginkgo biloba leaves. NSCLC = non-small cell lung cancer.

The targets of Ginkgo biloba leaves and NSCLC were imported into Venny 2.1.0, and a total of 109 intersection targets for Ginkgo biloba leaves against NSCLC were obtained, as shown in Figure 1. The targets are listed in Table 1, and the active ingredients against NSCLC are listed in Table 2.

**Table 1:** Potential targets of Ginkgo biloba leaves in treating NSCLC.

NO	Gene name	NO	Gene name	NO	Gene name	NO	Gene name
1	NOS2	29	CASP8	57	MMP2	85	MYC
2	PTGS1	30	PRKCA	58	MMP9	86	GJA1
3	ESR1	31	TGFB1	59	MAPK1	87	IL1B
4	AR	32	MAP2	60	RB1	88	CCL2
5	PPARG	33	IKBKB	61	CDK4	89	CXCL8
6	PTGS2	34	AKT1	62	IL6	90	HSPB1
7	KDR	35	TNF	63	TP53	91	SERPINE1
8	ESR2	36	MAPK8	64	NFKBIA	92	PTEN
9	MAPK14	37	MMP1	65	TOP1	93	IL1A
10	GSK3B	38	STAT1	66	MDM2	94	MPO
11	CDK2	39	CDK1	67	PCNA	95	ABCG2
12	PIM1	40	HMOX1	68	ERBB2	96	NFE2L2
13	TOP2A	41	CYP3A4	69	MCL1	97	NQO1
14	CCNA2	42	CYP1A2	70	BIRC5	98	PARP1
15	CCND1	43	CYP1A1	71	IL2	99	CHEK2
16	IL10	44	ICAM1	72	CCNB1	100	CRP
17	CYP2B6	45	VCAM1	73	IFNG	101	CHUK
18	UGT1A1	46	NR1I2	74	IL4	102	SPP1
19	PIK3CG	47	CYP1B1	75	XIAP	103	RASSF1
20	CHEK1	48	ALOX5	76	MET	104	E2F1
21	RELA	49	GSTP1	77	FOS	105	IGFBP3
22	PGR	50	GSTM1	78	EGF	106	IGF2
23	OPRM1	51	RXRA	79	CDKN2A	107	IRF1
24	BCL2	52	PLAU	80	POR	108	ERBB3
25	BAX	53	EGFR	81	RAF1	109	RASA1
26	CASP9	54	VEGFA	82	HIF1A		
27	JUND	55	BCL2L1	83	HSPA5		
28	CASP3	56	CDKN1A	84	CAV1		

NSCLC = non-small cell lung cancer.

**Table 2:** Potential ingredients of Ginkgo biloba leaves in treating NSCLC.

MOL ID	Name of active ingredients	OB (%)	DL
MOL001558	sesamin	56.55	0.83
MOL000492	(+)-catechin	54.83	0.24
MOL000096	(-)-catechin	49.68	0.24
MOL000354	isorhamnetin	49.6	0.31
MOL000098	quercetin	46.43	0.28
MOL007179	Linolenic acid ethyl ester	46.1	0.2
MOL000449	Stigmasterol	43.83	0.76
MOL001494	Mandenol	42	0.19
MOL000422	kaempferol	41.88	0.24
MOL011594	Isogoycyrol	40.36	0.83
MOL005043	campest-5-en-3beta-ol	37.58	0.71
MOL005573	Genkwanin	37.13	0.24
MOL000358	beta-sitosterol	36.91	0.75
MOL011604	Syringetin	36.82	0.37
MOL000006	luteolin	36.16	0.25
MOL003044	Chryseriol	35.85	0.27

NSCLC = non-small cell lung cancer.

### 3.4. Construction of drug-compound-target network

**Figure 2.**
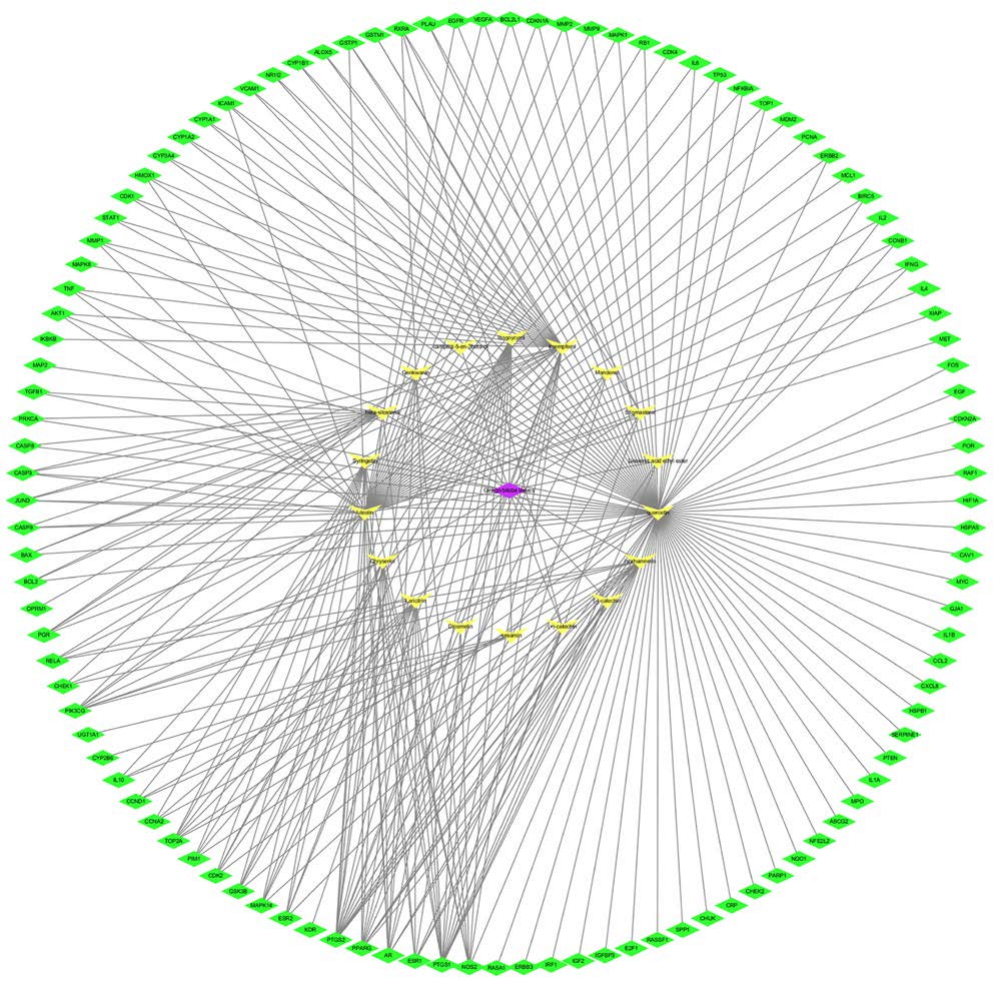
The “drug-compound-target” network of Ginkgo biloba leaves in the treatment of NSCLC. The purple diamond was utilized to represent the drug, the yellow color of V-shapes was used to represent the ingredients, and the green diamonds were used to represent the gene targets. NSCLC = non-small cell lung cancer.

The drug, ingredients, and targets were imported into the Cytoscape3.7.1 software. As shown in Figure 2, the purple diamond was utilized to represent the drug, the yellow color of V-shapes was used to represent the ingredients, and the green diamonds represented the gene targets. The top 3 core compounds of Ginkgo biloba leaves were quercetin, luteolin, and kaempferol according to the degree value of the network.

### 3.5. Construction of PPI network

**Figure 3.**
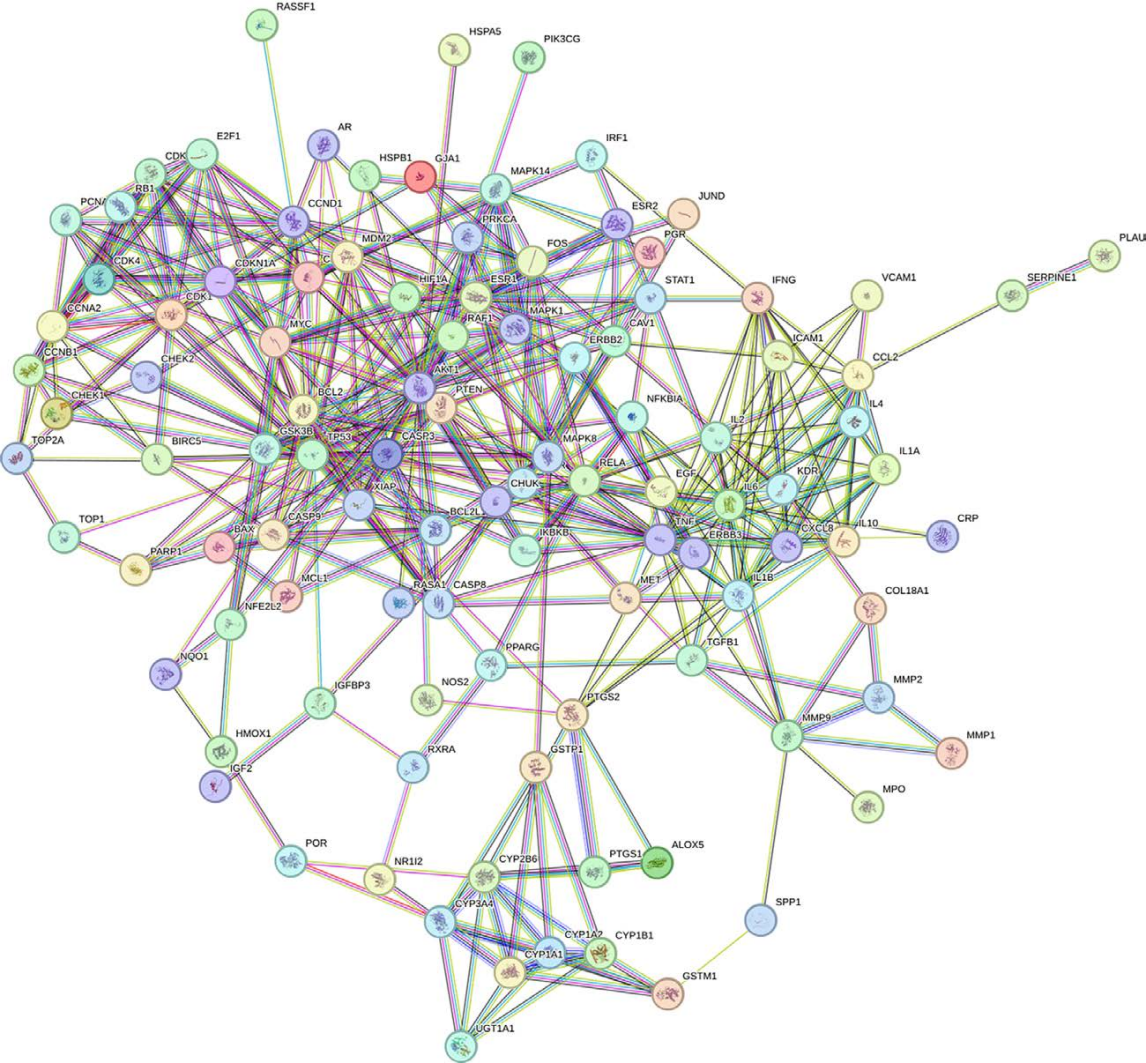
The PPI network of Ginkgo biloba leaves in treating NSCLC. NSCLC = non-small cell lung cancer, PPI = protein-protein interactions,.

**Figure 4.**
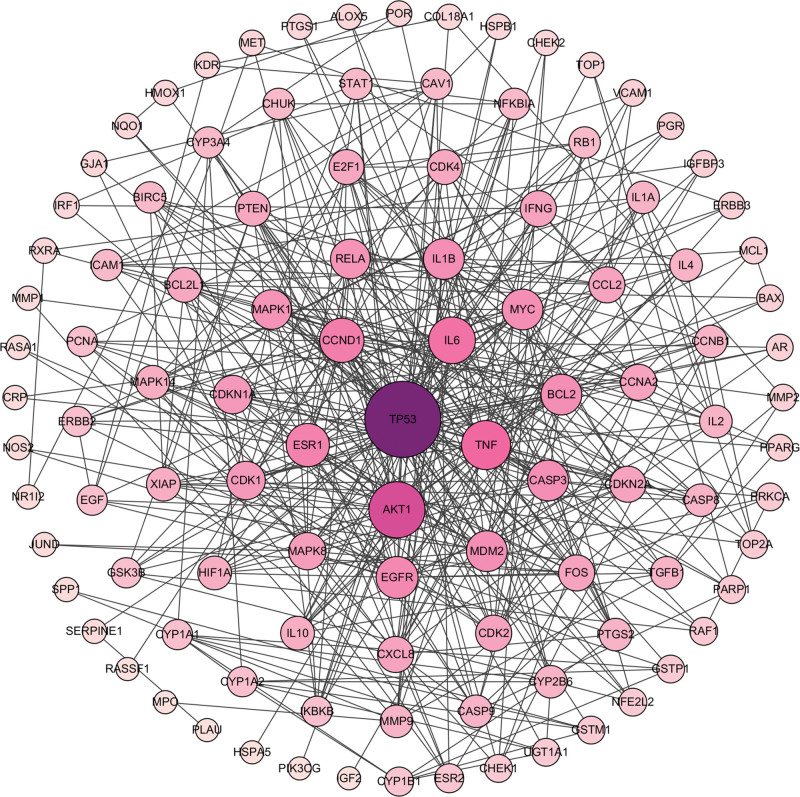
The PPI network of Ginkgo biloba leaves in treating NSCLC. The PPI network was selected by the Cytoscape 3.7.1 software. The nodes in the center have the highest degree of interaction. NSCLC = non-small cell lung cancer, PPI = protein-protein interactions.

A total of 109 intersection targets were imported into the STRING database to construct the PPI network. The species was set as “Homo Sapiens,” and the disconnected targets were hidden, as shown in Figure 3. Then, the TSV format of the PPI network was imported into the Cytoscape 3.7.1 software for topology analysis and visualization. According to the degree value, the key gene targets in treating NSCLC were TP53, AKT1, and TNF (Fig. 4).

### 3.6. GO and KEGG analysis

**Figure 5.**
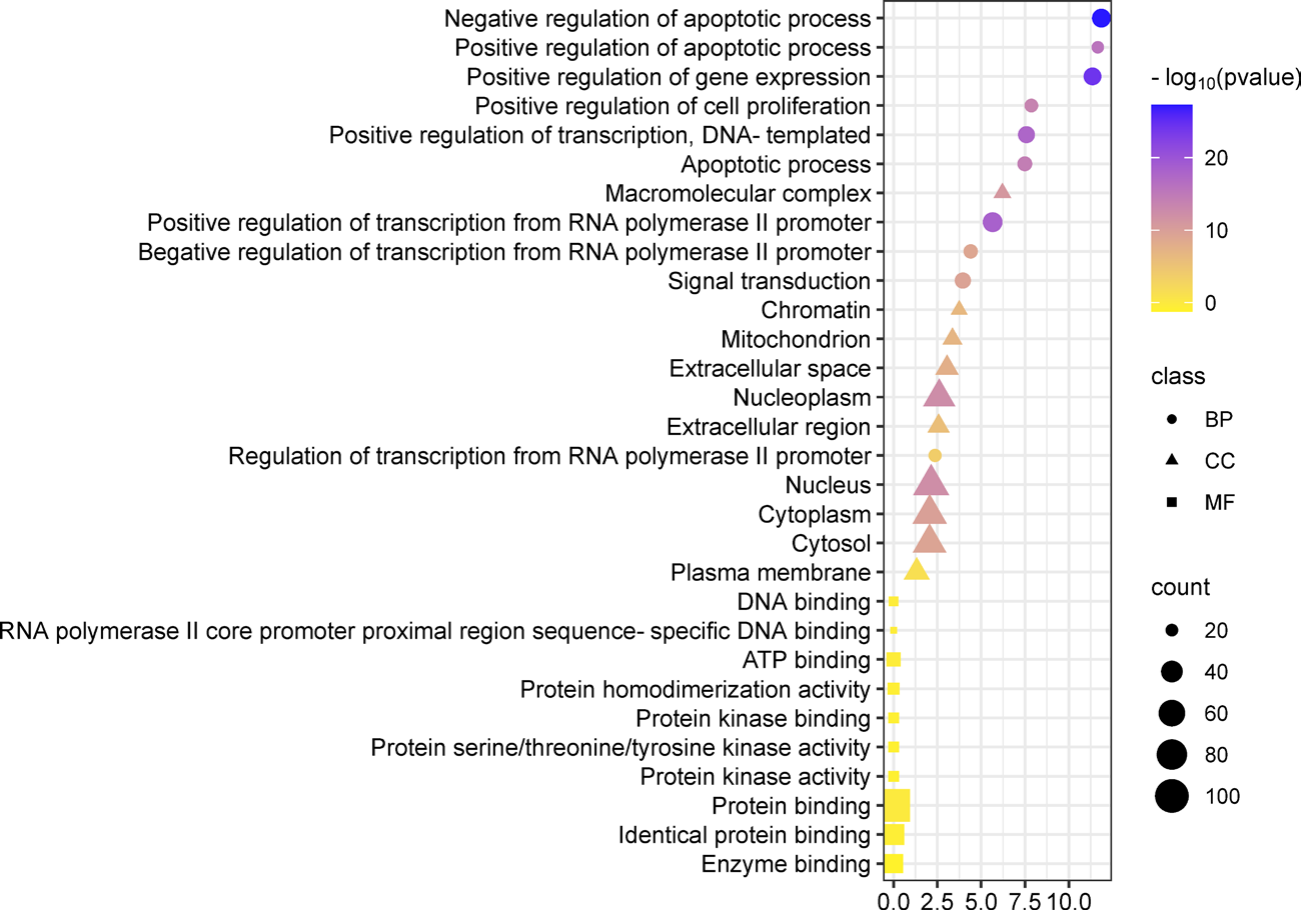
GO enrichment analysis of key targets.

**Figure 6.**
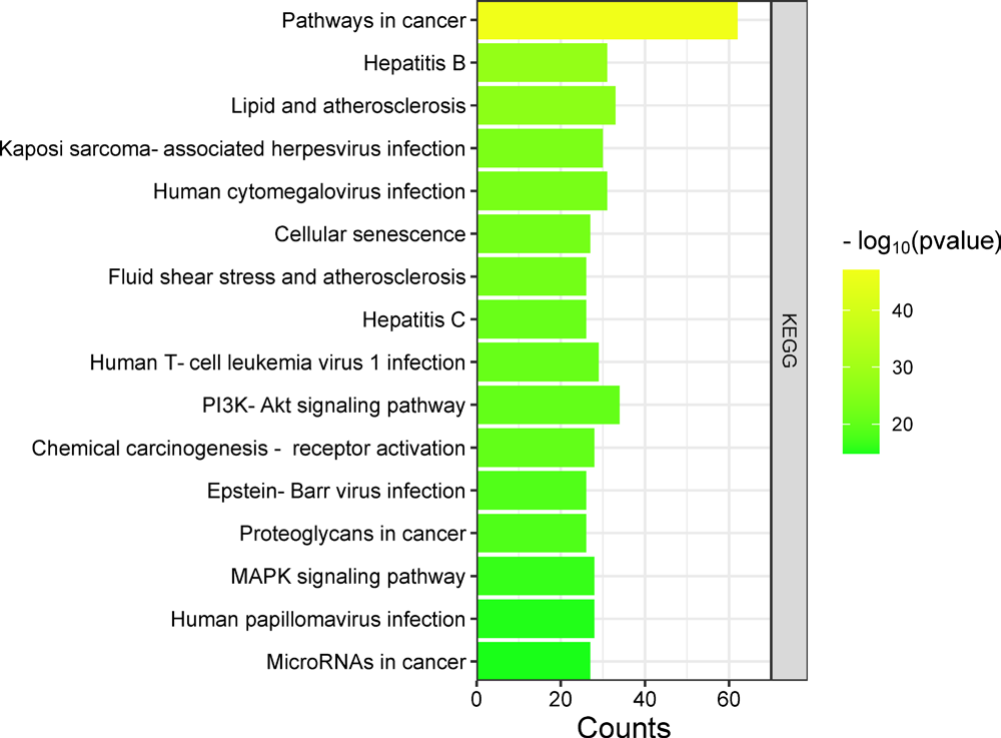
KEGG enrichment analysis.

The 109 interaction targets of Ginkgo biloba leaves and NSCLC were imported into the DAVID database for bioinformatic analysis. A total of 685 biological processes (BP), 67 cellular components (CC), 127 molecular functions (MF), and 160 KEGG pathways were obtained. The biological processes of potential targets mainly included negative regulation of apoptotic process, positive regulation of gene expression, positive regulation of transcription, DNA-templated, and positive regulation of transcription from RNA polymerase II promoter. The cellular components of potential targets mainly included nucleoplasm, nucleus, cytoplasm, and cytosol. Targets related to molecular functions mainly included protein binding, identical protein binding, and enzyme binding (Fig. 5). We selected the top 16 ranked KEGG analysis results for visualization, including PI3K-Akt and MAPK signaling pathways, which were closely associated with tumors (Fig. 6).

### 3.7. Molecular docking

**Figure 7.**
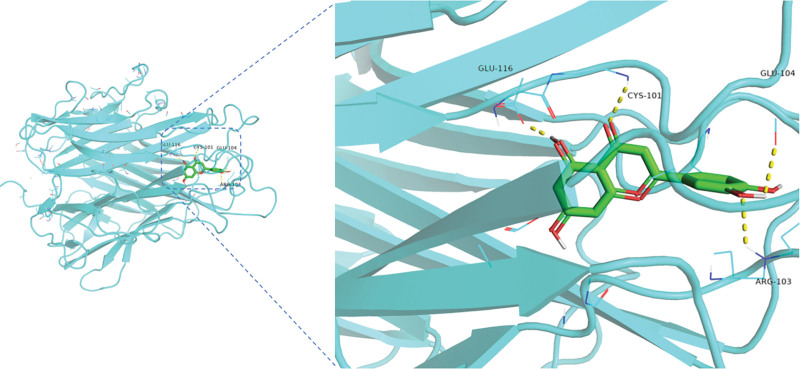
Display of partial molecular docking result.

The top 3 ranked components and targets were used for molecular docking, quercetin, luteolin, and kaempferol were picked from the drug-compound-target network, TP53, AKT1, and TNF were selected from the PPI network. A docking score less than or equal to −5.0 kcal/mol indicates a strong affinity between the docked compound and the target, among them, the docking binding energy between TNF and luteolin was the lowest (−8.6 kcal/mol), indicating that TNF and luteolin might be the core target of Ginkgo biloba leaves in the treatment of NSCLC, the binding energy were shown in Table 3. The results are shown in Figure 7.

**Table 3:** Docking results between key components and key targets.

Component	Target	Binding energy (kcal/mol)
Quercetin	AKT1	−6.4
	TNF	−7.1
	TP53	−7.4
Luteolin	AKT1	−7.0
	TNF	−8.6
	TP53	−7.3
Kaempferol	AKT1	−6.0
	TNF	−7.2
	TP53	−7.4

AKT1 = protein kinase B, TNF = Tumor necrosis factor, TP53 = tumor antigen p53.

## 4. Discussion

Lung cancer is a common malignant tumor in clinical practice, although there are various treatment methods such as surgery, radiotherapy, and chemotherapy, the mortality rate is still high. There are a huge number of active components found in TCM, and many components in TCM have been shown to inhibit NSCLC, however, the clinical effects are still limited. Ginkgo biloba leaves, have been clinically used for the treatment of some malignant tumors for many years, but we know little about its molecular mechanism in NSCLC, therefore, we expect to discover its potential targets, pathways for the treatment of NSCLC utilizing network pharmacology techniques, and to develop some drugs for the treatment of NSCLC.

TP53 is a tumor suppressor gene,^[26]^ which is widely altered in various solid tumors like NSCLC, TP53 alterations were associated with faster evolution resistance, proliferation, migration, and invasion.^[27,28]^ In lung squamous cell carcinoma patients, the derived TP53-associated signature is a specific prognostic biomarker and could provide therapeutic targets for the development of novel therapies.^[29]^ The PI3K-AKT pathway plays an essential role in the regulation of cell motility, invasion, and metastasis, AKT is known to facilitate metastasis,^[30]^ Mutant AKT1 promotes the self-renewal, tumorigenic potential of tumor initiating cells in immortalized human bronchial epithelial cells, and AKT inhibition inhibits these activities in primary NSCLC cells.^[31]^ IL-6, also known as interleukin-6, is a pleiotropic cytokine with important functions in the human immune system such as defense against pathogens, however, numerous studies have shown that the IL-6 pathway involved in inflammation and cancer, some anti-IL-6 drugs have demonstrated promising results in clinical trials.^[32]^ Liu et al^[33]^ found that IL-6 facilitates metastasis by up-regulating T-cell immunoglobulin domain and mucin domain 4 via NF-κB. Cytokines such as TNF-α in the tumor microenvironment may be closely related to tumor growth, metastasis, and prognosis, TNF-α was used clinically as a target to treat tumors.^[34]^ TNF exerts complex effects on the NSCLC, TNF and its receptors are widely expressed in NSCLC, and though TNF can promote cell death in neoplasms and has been utilized to treat some cancers, it may play an oncogenic role in NSCLC.^[35]^ CCND1 is overexpressed in NSCLC and is associated with tumorigenesis and progression.^[36]^ The down-regulation of CCND1 inhibits cell proliferation and promotes apoptosis of NSCLC,^[37]^ the inhibition of CCND1 enhances the anti-tumor effects of some targeted drugs in non-small cell lung cancer.^[38]^ Some herbal medicines have been shown to inhibit NSCLC growth by suppressing CCND1, for instance, Puerarin inhibits CCND1 expression by regulating miR-342/CCND1 axis.^[39]^

Quercetin is a bioflavonoid that exerts its anti-tumor effects on various neoplasms including NSCLC, it enhances the radiosensitivity of NSCLC cells via regulating miR-16-5p/WEE1 axis.^[40]^ Quercetin was shown to inhibit NSCLC in a variety of ways, including inhibiting the survival, proliferation, migration, and invasion of NSCLC.^[41]^ Luteolin is a flavonoid abundant in fruits and vegetables, study has proved that it can improve anti-tumor immunity in KRAS-mutant lung cancer,^[42]^ and inhibit the migration and invasion of NSCLC through MAPK and PI3K/Akt pathways.^[43]^ Kaempferol, a flavonoid from natural plant sources, has proved to be effective in anti-NSCLC, specifically in inducing apoptosis in NSCLC cells,^[44]^ promoting autophagy in NSCLC cells,^[45]^ increasing the sensitivity to radiotherapy,^[46]^ and inhibiting migration of A549 cells.^[47]^

The PI3K-AKT signaling pathway is a key signaling pathway that regulates the progression of metastasis,^[48]^ cell proliferation, differentiation, and metabolism.^[49]^ It has been shown that some natural substances like Fucoxanthin could inhibit proliferation, and arrest cell cycle at the G0/G1 phase in NSCLC cells through the PI3K-AKT signaling pathway.^[50]^ Tetrahydrocurcumin, which is isolated from Curcuma wenyujin, was found to induce autophagy in human A549 cells via the PI3K-AKT signaling pathway.^[51]^ Accordingly, we conclude that Ginkgo biloba leaves could act on NSCLC through multiple signaling pathways, and be associated with the inhibition effect of cell proliferation and promoting apoptosis, etc.

There are still some limitations in the present study, for instance, there are some active ingredients with no targets in the TCMSP database that fulfill both OB ≥ 30% and DL ≥ 0.18, such as ginkgolide B and ginkgolide C. In addition, complex chemical reactions will happen when decocting medicine, and some elements may become invalid. Moreover, network pharmacology combined with biological experiments can reveal the mechanism of TCM more comprehensively. Thus, in the future, we will conduct target prediction and further experiments of some components to verify deeper mechanisms of Ginkgo biloba leaves against lung cancer.

## 5. Conclusion

Based on network pharmacology and molecular docking techniques, we confirmed that Ginkgo biloba leaves against NSCLC through multi-compounds, multi-targets, and multi-pathways, and preliminarily elucidated the potential mechanism related to Ginkgo biloba leaves in the treatment of NSCLC. In this study, we systematically analyzed the key components, gene targets, protein interactions, biological functions, signaling pathways, and possible mechanism of Ginkgo biloba leaves. Quercetin, luteolin, and kaempfero were screened as the major compounds in Ginkgo biloba leaves. The main targets of Ginkgo biloba leaves in the treatment of NSCLC were TP53, AKT1, TNF, IL-6, and CCND1, we conclude that Ginkgo biloba leaves may act through PI3K-AKT and MAPK signaling pathways. In the future, we will continue to follow the relevant progress in this field and conduct in vivo and in vitro experiments to verify the underlying mechanism of Ginkgo biloba leaves against NSCLC.

## Acknowledgments

The authors gratefully acknowledge the financial support by The Project of National Famous Traditional Chinese Medicine Practitioner Zhusheng Zhu Studio (No. 2022-75).

## Author contributions

**Conceptualization:** Mingxiao Wang.

**Data curation:** Mingxiao Wang.

**Funding acquisition:** Xun Zhou.

**Investigation:** Mingxiao Wang.

**Software:** Mouqi Bai.

**Visualization:** Mingxiao Wang.

**Writing – original draft:** Ruochen Li.

**Writing – review & editing:** Xun Zhou.
